# The History of Social and Behavioral Research in Gerontology

**DOI:** 10.1093/geroni/igaf122.930

**Published:** 2025-12-31

**Authors:** Soomi Lee, Karl Pillemer

## Abstract

Celebrating GSA’s 80th anniversary, we launched the inaugural session of “History of Gerontology” at GSA 2024, which generated significant interest and enthusiasm among members and attendees. The session highlighted the importance of continuing this initiative to provide an educational and scholarly exchange platform rooted in historical perspectives. Building on this momentum, we propose a second symposium in this series, focusing on the history and importance of social and behavioral research in gerontology. This theme aligns well with this year’s focus, “Innovative Horizons in Gerontology,” fostering discussions on how advancement in social and behavioral research can generate novel insights into disease prevention and well-being promotion. While social and behavioral research in gerontology encompasses many significant topics, our three panelists will focus on their expertise in long-term care, caregiving, and stress research, sharing valuable insights and experiences. Dr. Kathleen Wilber will reflect on lessons from over five decades of long-term care program and policy development. Dr. Steven H. Zarit will discuss the evolution of caregiving research and the challenges of capturing the complexity of caregiving. Dr. David Chiriboga will examine the history of stress research and its contributions to advancing gerontology. The discussant, Dr. Karl Pillemer, will engage panelists and the audience in exploring the role of social and behavioral research in Translational Geroscience. This symposium provides an opportunity for the Academy for Gerontology in Higher Education (AGHE) and the broader GSA community to reflect on the lasting impact of social and behavioral research in enhancing support for the aging population. This is a collaborative symposium between the Age Inclusivity in Higher Education and Generativity and Aging Interest Groups.

